# Breast cancer as an example of tumour heterogeneity and tumour cell plasticity during malignant progression

**DOI:** 10.1038/s41416-021-01328-7

**Published:** 2021-04-06

**Authors:** Fabiana Lüönd, Stefanie Tiede, Gerhard Christofori

**Affiliations:** Department of Biomedicine, University of Basel, Basel, Switzerland

**Keywords:** Metastasis, Epithelial-mesenchymal transition

## Abstract

Heterogeneity within a tumour increases its ability to adapt to constantly changing constraints, but adversely affects a patient’s prognosis, therapy response and clinical outcome. Intratumoural heterogeneity results from a combination of extrinsic factors from the tumour microenvironment and intrinsic parameters from the cancer cells themselves, including their genetic, epigenetic and transcriptomic traits, their ability to proliferate, migrate and invade, and their stemness and plasticity attributes. Cell plasticity constitutes the ability of cancer cells to rapidly reprogramme their gene expression repertoire, to change their behaviour and identities, and to adapt to microenvironmental cues. These features also directly contribute to tumour heterogeneity and are critical for malignant tumour progression. In this article, we use breast cancer as an example of the origins of tumour heterogeneity (in particular, the mutational spectrum and clonal evolution of progressing tumours) and of tumour cell plasticity (in particular, that shown by tumour cells undergoing epithelial-to-mesenchymal transition), as well as considering interclonal cooperativity and cell plasticity as sources of cancer cell heterogeneity. We review current knowledge on the functional contribution of cell plasticity and tumour heterogeneity to malignant tumour progression, metastasis formation and therapy resistance.

## Background

Breast cancer is a highly heterogeneous disease that can be caused by a variety of distinct genetic alterations in mammary epithelial cells, leading to vastly different disease manifestations in individual patients. A combinatorial evaluation of the histopathology of the primary tumour and of the expression pattern of hormone receptors (oestrogen and/or progesterone receptors; ER/PR) and epidermal growth factor receptor 2 (HER2/Neu), together with further genomic and transcriptomic profiling, classifies breast cancer as one of the following molecular subtypes: normal breast-like, luminal A (ER+/PR+ and Ki67-low), luminal B (ER+/PR+ and HER2+ or HER2–, and Ki67-high), HER2-enriched (HER2+), basal-like and claudin-low.^1–8^ These patient-to-patient differences, which constitute ‘intertumoural heterogeneity’, largely affect patient prognosis and treatment options.^9^ Alongside these patient-to-patient differences, enormous diversity can exist in tumour cell subpopulations within a patient’s primary tumour and individual metastases—this is referred to as ‘intratumoural heterogeneity’.

**Fig. 1:**
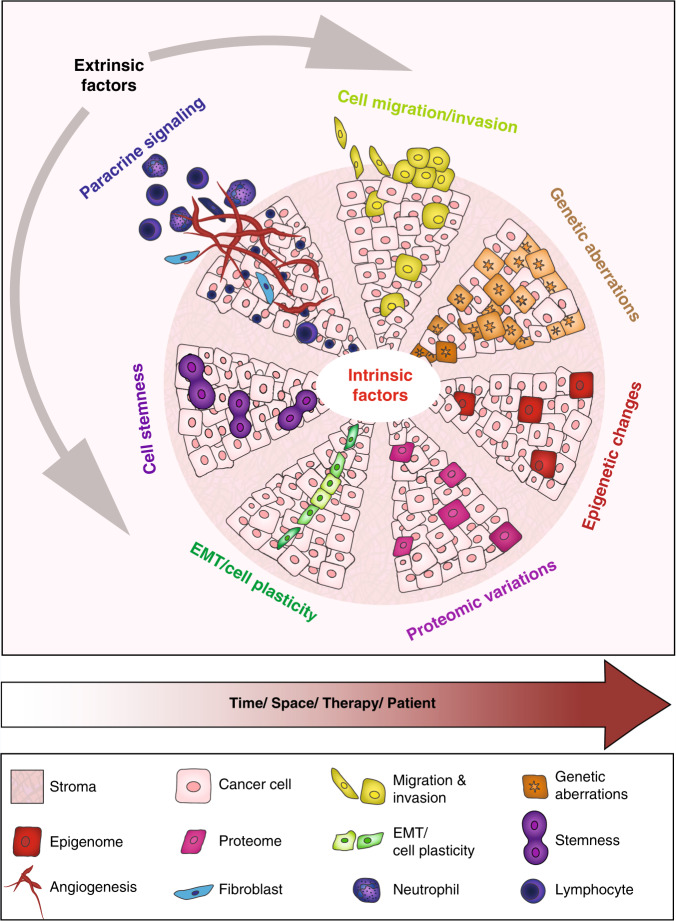
Intratumoural heterogeneity as a consequence of cancer-cell-intrinsic and cancer-cell-extrinsic factors. Individual cancer cells can differ with respect to a large number of capabilities, which are regulated at different levels by cell-intrinsic cues, including genetic, epigenetic and proteomic changes. The major intrinsic causes of intratumoural heterogeneity include cancer cell migration and invasion, genetic instability, epigenomic/transcriptomic and proteomic regulation, the degree of epithelial-to-mesenchymal transition (EMT) and cell plasticity, and the extent of stemness. Extrinsic factors in the tumour microenvironment (TME), such as the tumour vasculature and residing and infiltrating cells of the immune system, also contribute to intratumoural heterogeneity through cell–cell interactions and paracrine signalling. Intratumoural heterogeneity evolves in time and space, changes in response to therapy and the development of resistance, and varies between different patients (adapted from ref. ^13^).

Generally speaking, inter- and intra-tumoural heterogeneity are a consequence of differences in a number of cancer-cell-intrinsic parameters, such as genetic profile, interplay between the genome, epigenome/transcriptome and proteome, migration and invasion capabilities, proliferation, stemness and intrinsic cell plasticity (Fig. 1). As well as these cell-autonomous traits, extrinsic, microenvironmental factors also contribute to tumour heterogeneity. These factors include tumour hypoxia and the extent of vascularisation, interactions of cancer cells with cells of the tumour stroma, such as endothelial cells, pericytes, fibroblasts, and the contribution of a variety of tumour-infiltrating cells of the innate and the adaptive immune systems. These complex interactions between cells and the extracellular matrix (ECM) were first conceptualised as dynamic reciprocity in 1982 by Bissell et al.^10^ These bidirectional interactions induce stochastic and dynamic changes during malignant cancer progression and, therefore, require cancer cells to constantly adapt to new environmental cues and challenges. The ability to rapidly react by genetic and epigenetic reprogramming appears to involve a cellular status of high plasticity, as will be described below.^2,11–15^

In addition to opening up new avenues for the reconstruction of the temporal and spatial evolution of cancer, genome sequencing has also provided novel insights into heterogeneity between tumours, even of the same pathological subtype. Additional technological advances in novel whole omics profiling also offer the opportunity to better comprehend the enormous complexity and interplay of the genetic, epigenetic, transcriptomic and proteomic variations that ultimately cause phenotypic heterogeneity (Fig. 1).^13,16^ In 2000, DNA microarray profiling demonstrated for the first time distinctive gene expression patterns for normal breast, luminal-like, HER2+ and basal-like subtypes, thus already highlighting the presence of considerable intertumoural heterogeneity;^6^ a claudin-low subtype that specifically expresses markers of epithelial-to-mesenchymal transition (EMT) and stemness, as well as stromal and immune-related signatures, was subsequently added to this classification.^7,8^ In 2002, microarray gene expression analysis of 117 breast tumours identified three expression signatures that were predictive of therapy response: a signature indicative of poor prognosis consisting of genes important for cell-cycle progression, invasion, metastasis and angiogenesis; an ER signature; and a signature that identifies carriers of BRCA1 mutations.^17^

Around 2010, a research goal has been to simultaneously sequence the genome and the transcriptome of human breast cancer samples, including single-cell sequencing.^18,19^ For example, Curtis and colleagues have described the genomic and transcriptomic landscape of 2000 human breast cancer samples by using an integrated analysis of copy number variations and gene expression patterns and they have identified additional subtypes of breast cancer.^20^ A similar analysis of triple-negative breast cancers (TNBC) have revealed a high heterogeneity within this one type of breast cancer, indicating that patients need to be analysed at an individual level.^21^ At around the same time, the Cancer Genome Atlas Network combined various methods to draw the molecular portraits of human breast cancers in a more holistic way, revealing a tremendous amount of variation even within individual breast cancer subtypes.^22^ In 2017, an analysis of somatic copy number alterations led to a newer classification of breast cancers into integrative clusters associated with prototypical patterns of single-nucleotide variants and distinct clinical outcome.^23^ Clinically relevant, these molecular classifications are associated with patient prognosis and possible treatment options. Hence, the genomic and transcriptomic analysis has to be refined for individual patients to match the observed high heterogeneity between tumours of individual patients even within one specific breast cancer subtype.

In this review, we summarise recent progress in the identification of the molecular and cellular determinants of tumour heterogeneity with a focus not only on genetic aberrations, but rather on epigenetic and transcriptomic differences defining specific tumour cell phenotypes and their functional contribution to malignant tumour progression. A major focus is given to the role of EMT and cell plasticity in generating intratumoural heterogeneity and the varying contributions to tumour cell invasion, metastasis formation and the development of therapy resistance.

## Tumour heterogeneity and clonal evolution

Whole genome and exome sequencing of human tumour samples has unveiled the existence of extensive intratumoural genetic heterogeneity (Fig. 1).^9,21,22,24^ This somatic heterogeneity ultimately results in the formation of subclones with different biological capabilities, which will follow Darwin’s principle of ‘survival of the fittest’. As early as 1976, Peter Nowell described the concept of clonal evolution, whereby subclones of cells with a growth advantage in a certain ecosystem will persist, survive and ultimately expand, whereas less fit subclones are unable to compete against the others and become extinct.^25,26^

### Temporal evolution of cell-intrinsic traits

Advances in DNA sequencing technologies eventually made it possible to infer the evolution of human tumours based on genomic alterations. For example, multi-region DNA sequencing of primary and metastatic pancreatic tumour samples allowed the reconstruction of distinct phylogenetic trees that describe clonal evolution in this type of cancer.^27,28^ Similarly, Nik-Zainal and co-workers reconstructed the evolutionary tracks of 21 breast cancer cases,^29,30^ demonstrating that, due to the early occurrence of driver mutations and alterations in cancer genes, genetic variation arises, selective pressures are shifted, and various subclones expand. Along these lines, DNA barcoding and massive parallel sequencing of samples from xenograft transplantation mouse models of human breast cancer confirmed the complex heterogeneity of clonal subpopulations: some subclones remain stable, some expand, and others vanish over time. As a result, dramatic changes in clone numbers and sizes and in the emergence of new subclones within and between primary and secondary tumours occur during breast cancer progression.^31–33^ Barcoding experiments have also revealed that the dominant clones of primary tumours do not necessarily contribute to tumour metastasis, distinguishing the cells that are shed from primary tumours but that do not yet form metastasis (shedders) from actual metastasis-seeding cells (seeders), indicating that therapeutic strategies need to target selected subpopulations.^34,35^

Data obtained from a variety of genomic profiling experiments of cancer cells from primary tumours and from distant sites subsequently allowed three distinct evolutionary patterns of primary tumour and metastasis to be inferred: a simple linear evolution model; a parallel evolution model with early metastatic spread; and a model assuming late seeding from either a single or multiple subclone(s) within the primary tumour.^36,37^ In addition, a more recent study based on topographical genomic copy number profiling of 1293 single cells from ten breast cancer patients with ductal carcinoma in situ has led to the proposal of a fourth, so-called ‘multi-clonal invasion’, model.^38^ Here, genomic evolution within the mammary ducts gives rise to multiple tumour subpopulations, which subsequently co-migrate into the underlying tissue to form an invasive carcinoma. Regardless of the evolutionary path, a tumour might take, genetic heterogeneity and subclonal diversification enhance a primary tumour’s robustness and ultimately complicate patient prognosis, therapy response and clinical outcome.

### Spatial evolution of cell-intrinsic traits

Cell-intrinsic traits not only evolve temporally throughout tumour progression, but also spatially within a primary tumour and its secondary lesions (Fig. 1).^39^ To investigate the topographical distribution of subclones, Yates and colleagues representatively sampled surgically excised invasive primary breast tumours in a multiple biopsy approach^41^. Most of the tumours exhibited at least one mutation that was confined to one to three neighbouring tissue regions, indicating a locally constrained expansion of subclones. Only a few tumours displayed clonal intermingling throughout the whole tumour tissue.^40^ In a similar study, when multifocal breast cancer lesions with comparable tumour grades and ER and HER2 status were subjected to whole genome and targeted sequencing,^41^ genetically similar lesions were topographically located closer to each other than genetically distinct lesions, indicating a process of clonal evolutionary outgrowth. Interestingly, despite histopathological homogeneity, considerable inter-lesion heterogeneity was found in one-third of the patients for oncogenic mutations in *PIK3CA*, *TP53*, *GATA3* and *PTEN*, the most frequent driver mutations in breast cancer.^41^ These results already indicate that the simple genomic analysis of a sample taken from an undefined topographical position might not adequately molecularly characterise a tumour.

## Cell plasticity as a source of tumour heterogeneity

The genetic, epigenetic, transcriptomic and proteomic traits of a cell are not fixed determinants. Triggered by stimuli from the microenvironment and by stochastic changes in gene expression, cancer cells can transition between phenotypic states through cell plasticity. This condition of cellular phenotypic uncertainty, yet high reactivity to external and internal cues, is often referred to as a state of high cell plasticity. It greatly contributes to tumour heterogeneity. The functional and molecular definition of cellular plasticity is context-dependent, yet cell plasticity is a broadly acceptable explanation of various biological and cancer-related processes.^42–44^

### Hybrid EMT states

EMT and its reverse process, mesenchymal-to-epithelial transition (MET), constitute particularly well-studied programmes of high cell plasticity, occurring, for example, during fundamental processes such as embryonic development.^45^ During EMT in cancer progression, epithelial cancer cells lose their cell–cell adhesions, dedifferentiate and acquire a migratory and invasive mesenchymal phenotype. This process has been associated with metastasis and drug resistance. Rather than being a binary switch, however, EMT/MET allows cells to transition between a spectrum of phenotypic hybrid states,^45–47^ although little is known about this heterogeneous spectrum, the stability of these cell states or their functional characteristics. Pastushenko and colleagues characterised six populations of distinct EMT states in transgenic mouse models of skin squamous cell carcinoma and of breast cancer.^48^ These EMT states exhibited different functional characteristics regarding migration, invasion, metastasis and stemness, although they all showed a similar tumour-propagating cell capacity. Interestingly, they also presented with different degrees of intrinsic cell plasticity, exemplified by their potential to spontaneously transition to another EMT state when subcutaneously transplanted or intravenously injected into mice. Notably, whereas only transitions between EMT hybrid states occurred in the subcutaneously transplanted primary tumours, cells in the lung could also revert back to an epithelial state,^48^ indicating that cancer cell plasticity is determined both by cell-intrinsic characteristics and the microenvironment.

Lineage-tracing studies in vivo and 3D imaging have provided a first glimpse into EMT dynamics during early and late stages of malignant tumour progression.^48,49^ A diverse selection of EMT markers is often used to assess EMT/MET in tumour progression and metastasis formation.^50,51^ However, although EMT markers and EMT transcription factors can be useful when adjusted to a specific cancer model, they have fallen short in capturing the dynamic nature of hybrid EMT states.^52^ Indeed, EMT is a process that is potentially inducible in different cell types and also differentiation stages.^45^ For instance, triple-negative breast cancer (TNBC) is considered to exemplify a highly de-differentiated, mesenchymal cellular phenotype, but TNBC cells can also undergo EMT-like changes and transiently enhance cellular plasticity, which, in turn, contributes to metastasis and drug resistance.^53,54^ Thus, a mesenchymal cancer cell can also be triggered to undergo further EMT-like changes, thereby displaying phenotypic plasticity. These observations raise the question of how hybrid, intermediate stages of EMT and a linear transition process of EMT can be reconciled. Can we identify a plasticity signature and capture a plasticity moment induced or manifested by reiterated processes of EMT and MET, and are these processes not linear at all?

Another source of tumour heterogeneity lies in the various degrees of cell plasticity that might be driven by the extent of (de)differentiation of cancer cells. The cancer stem cell (CSC) model proposes that tumour growth and progression are driven by CSCs, which have the ability to self-renew and differentiate, thereby fostering the bulk of tumour cells. Mounting evidence supports the idea that CSCs might dynamically arise by the dedifferentiation of ‘regular’ cancer cells. In particular, EMT has been linked to the generation of breast CSCs.^55–58^ However, stemness is not a unique feature of the mesenchymal phenotype and plasticity between differentiated epithelial cancer cells and epithelial CSCs has also been observed.^59,60^ Similarly, breast cancer cells can transition between luminal, basal and progenitor-like cell states,^59,61–63^ further underscoring the potential of cell plasticity to affect cancer cell phenotype and malignancy and thus also increasing the complexity of clinical care.

### What causes intrinsic cell plasticity?

The roots of intrinsic cell plasticity are poorly understood. Studies of breast cancer indicate that the extent to which cancer cells undergo EMT might be dependent on the mutational landscape. For example, larger numbers of tumour cells that had undergone EMT were found in MMTV-Myc-driven mammary tumours as compared with MMTV-PyMT-driven or MMTV-Neu-driven mammary tumours. This increased number might be due to the pleiotropic oncogenic functions of c-Myc as compared with the single pathway activations by polyoma middle T (PyMT) or the Neu oncogene;^64^ indeed, similar to c-Myc the introduction of an oncogenic PIK3CA mutation into mammary epithelial cells of basal or luminal lineage induced multipotency and led to the formation of heterogeneous multi-lineage tumours.^62,63^ However, the tumours were phenotypically different, depending on whether they originated from a basal or a luminal cell: transformed basal cells mainly formed benign differentiated tumours, whereas transformation of luminal cells induced aggressive tumours.^62,63^ These data indicate that the epigenetic landscape of the originating cancer cell might have a long-lasting effect on its (de)differentiation potential and plasticity.

## Intratumour heterogeneity and cellular interactions

A heterogeneous population of cells facilitates the interaction of diverse subpopulations of cancer cells with each other—predominantly through interclonal cooperativity rather than competition—and with cells in the tumour microenvironment (TME) by physical interaction or via paracrine signalling networks to drive cancer progression and metastasis. The studies described below offer a glimpse into the interclonal cooperativity that occurs between breast cancer subclones as well as between breast cancer cells and stromal cells and the TME, and the impact of these interactions on malignant breast cancer progression. However, many more critical interactions of cancer cells exist—for instance, with other types of immune cell and cells of the vascular system that contribute to tumour progression; these interactions have been reviewed elsewhere (Fig. 1).^65^

### Interclonal cooperativity

Tumour heterogeneity is frequently manifested by interclonal cooperativity or dependency. For example, in a transgenic mouse model of Wnt1-driven mammary tumorigenesis (MMTV-Wnt1), a heterogenous mixture of basal and luminal cancer cells demonstrates interclonal cooperation to support their own tumour growth. Despite the initial expression of Wnt1 by bipotent progenitor cells, these produce basal and luminal cells with luminal tumour cells expressing Wnt1, while basal and luminal cells gained *HRas* mutations. The cancer-driving *HRas*-mutant basal cells were deficient for Wnt signalling, but recruited the *HRas*-wildtype luminal cells to secrete Wnt1 to drive biclonal primary tumour growth.^66,67^ However, breast cancer subclones can cooperate not only with neighbouring clones in the same primary tumour, but also with cancer cells in metastatic lesions. Kim et al.^68^ identified a bidirectional spread of metastatic cancer cells from the primary tumour to the metastatic sites as well as from the metastatic lesions back to the primary tumour. This so-called ‘self-seeding’ does not require further adaptation by the tumour cells and appears to be directed by the interleukin (IL)-6/8-mediated attraction of circulating tumour cells (CTCs) from primary tumours and metastases back to primary tumours. It might promote tumour growth, angiogenesis and stromal recruitment.^68^ Similarly, whole genome sequencing of matched primary and metastatic prostate cancer samples has revealed interclonal cooperation between subpopulations of cells of primary tumours and metastatic lesions and the polyclonality of metastatic lesions at different metastatic sites,^69^ i.e. metastasis may also present high tumour heterogeneity and interclonal cooperativity within a metastatic lesion. As further described below, breast cancer cells with luminal and basal epithelial phenotypes, and possibly epithelial/mesenchymal features, might disseminate collectively, giving rise to polyclonal metastases.^70,71^

### Tumour–stroma interactions

As well as homotypic interactions between cancer cells themselves, extensive heterotypic cooperation of cancer cells with various types of cell in the TME, including endothelial cells, fibroblasts and cells of the immune system, occurs. These interactions jointly dictate the pace of tumour progression, the occurrence of metastasis and a patient’s therapy response (Fig. 1).^72–76^ Conversely, even a minor subpopulation of cancer cells might drive tumour growth by inducing microenvironmental changes^77^—for example, by activating angiogenesis or by recruiting fibroblasts or cells of the immune system to the primary TME or by changing the composition and/or the physicochemical consistency of the TME. These bidirectional heterotypic interactions between cancer cells and the cells of the TME are critical for tumour progression.^73^

In particular, interactions between cancer cells and cancer-associated fibroblasts (CAFs) during breast cancer metastasis have been well studied. For example, fibroblast-derived caveolin-1 causes increased fibroblast contraction, matrix alignment and stiffening of the microenvironment, which, in turn, promotes breast cancer cell elongation, directional migration, and metastasis.^78^ As further discussed below, fibroblasts might also act as leaders in cancer cell migration and invasion.^79–81^ Finally, cooperation between fibroblasts and breast cancer cells might contribute to metastatic colonisation.^82^ Here, infiltrating CSCs induce fibroblasts to secrete periostin, an ECM protein, which promotes metastatic colonisation by enhancing Wnt signalling in the tumour cells.

**Fig. 2:**
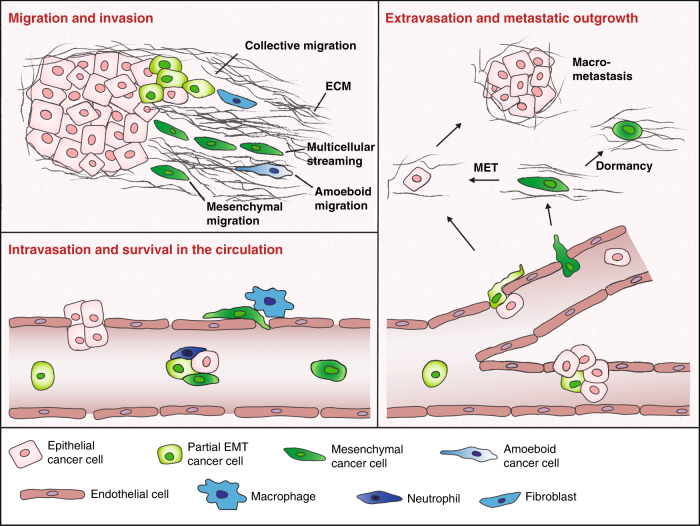
Epithelial-to-mesenchymal plasticity during the metastatic process. Cancer cells can invade the surrounding tissue as single cells by epithelial-to-mesenchymal transition (EMT) followed by protease-independent amoeboid migration, or by EMT and protease-dependent mesenchymal migration. Cells in a hybrid EMT state can migrate as cohesive groups that are often led by a mesenchymal cell (a cancer cell or fibroblast), while mesenchymal cancer cells migrate as single cells or as multicellular streams. Cancer cells can actively intravasate or be passively shed into the circulation. Perivascular macrophages can aid cancer cells in the intravasation process. Circulating tumour cells (CTCs) are found in various stages of EMT as single cells or in clusters with epithelial, hybrid EMT and mesenchymal cancer cells as well as stromal cells, such as neutrophils. Successful metastatic outgrowth requires an epithelial phenotype, which most likely arises from invasive mesenchymal cancer cells undergoing mesenchymal-to-epithelial transition (MET), while tumour dormancy might be associated with a mesenchymal phenotype. Note that the molecular details of the differential regulation of the distinct modes of cancer cell migration and invasion, the intravasation process, the decision to circulate either as single cancer cell or to form CTC clusters, and CTC extravasation, tumour dormancy and metastatic outgrowth, are still widely unknown and remain to be explored.

Tumour-associated macrophages (TAMs) have also been shown to contribute to malignant tumour progression at multiple levels. Macrophages are, for example, recruited by the chemokine ligand 2 (CCL2) secreted by cancer cells and, once at the tumour site, they are reprogrammed from an M1 anti-tumour phenotype to an M2 pro-tumorigenic phenotype, which promotes the secretion of pro-angiogenic, pro-invasive and immunosuppressive factors.^83–85^ TAMs can also induce early dissemination from the primary tumour by producing Wnt1^86^ and play a critical role in guiding the intravasation of invasive cancer cells into the bloodstream^87^ (Fig. 2).

In addition to the importance of the cooperativity of breast cancer cells with fibroblasts, their interaction with neutrophils has been shown critical for the formation of metastasis. RNA sequencing analysis of polyclonal metastatic breast tumours revealed that cancer cell subclones expressing IL-11 and vascular endothelial growth factor (VEGF)-D systemically prime pulmonary stromal cells to secrete neutrophil-stimulating factors. This leads to the attraction of pro-tumorigenic neutrophils, which enhance the ability of breast cancer cells to disseminate.^88,89^ Interestingly, the physical association of neutrophils and breast cancer cells in heterotypic CTC clusters drives cell-cycle progression in the circulation and enhances metastatic seeding.^90^ Thus, the interaction of breast cancer cells and neutrophils contributes to the metastatic process at multiple stages (Fig. 2).

Major progress has been made in investigating how cancer cells escape immune surveillance during malignant progression. In fact, it appears that programmes underlying cancer cell malignancy, such as EMT, also include various molecular changes that support immune tolerance, an observation that is important for improving current immunotherapies^91,92^ Notably, in some tumours there is an increase in immune cell infiltrations (‘hot tumours’), while in others immune cells are actively repressed from infiltrating the TME (‘cold tumours’) by the downregulation of immune cell-attracting cues and homing receptor systems. Most importantly, tumours might escape immunosurveillance by activating checkpoint blockades by the expression of PD1, PD-L1, CTLA4 and others by tumour cells and immune cells to prevent the activation of cytotoxic T cells for tumour cell killing. These molecules are the targets of current immunotherapies.^93^ Finally, non-cellular cues from the TME, including tumour hypoxia or changes in the stiffness of the ECM, critically affect cancer cell behaviour and phenotypes. These changes in the physicochemical parameters of the TME have been repeatedly shown to support malignant tumour progression and to play a critical role in increasing cell plasticity during malignancy and thus in tumour heterogeneity.^94^

## Tumour heterogeneity and metastasis formation

Whole omics approaches have been increasingly employed to investigate the functional implications of tumour heterogeneity in malignant progression and metastasis. A comparison of gene expression profiles of primary tumours and secondary lesions from different metastatic sites revealed tremendous heterogeneity, which was linked to organotropism. In vivo selection and transcriptomic analysis of bone- and lung-homing breast cancer cell lines have identified numerous genes that are crucial for specific organotropism and site-specific outgrowth in bone, lung and other organs.^95,96^ Similarly, single-cell analysis of early and late metastatic breast cancer cells has revealed dramatic differences in gene expression: whereas early metastatic cells presented with a basal/stem cell-like signature and expressed genes associated with a de-differentiated, EMT-like phenotype, cells in established metastases showed a more differentiated, luminal-like and proliferative signature.^53^ Whole exome sequencing of matched primary tumour and metastasis from individual patients with a variety of cancer types, including of the breast, revealed that polyclonal seeding occurs frequently in untreated patients, while it is rare in therapy-treated patients. Hence, specific mutations that might already exist in early primary tumours appear to be selected for in metastasis by therapy, although they might not contribute to metastatic dissemination and outgrowth as such^97^ and might instead be associated with drug resistance (see below).

Obradovic and colleagues^98^ undertook transcriptomic, proteomic and phospho-proteomic profiling of primary tumours and their corresponding metastases in transgenic mouse models of breast cancer. Their data revealed an increase in stress hormones during tumour progression, which resulted in the activation of the glucocorticoid receptor specifically at metastatic sites—this heterogenic expression of glucocorticoid receptor increased tumour heterogeneity and was critical for metastatic colonisation.^98^

Considered to be the precursors of metastases, CTCs have become a major focus of cancer research for the study of tumour heterogeneity and plasticity. Notably, CTC clusters transpire to be more efficient in metastatic seeding and outgrowth than do single CTCs.^99^ Whole genome bisulphite sequencing in combination with RNA sequencing has revealed differences in the epigenetic landscape of single CTCs versus CTC clusters. Of note, CTC clusters exhibited active (hypomethylated) transcriptional binding sites in embryonic stem cell genes, which probably contributes to their enhanced metastatic potential.^100^

Taken together, the emergence of whole omics techniques has led to an increasing understanding of tumour heterogeneity and its functional implications, especially in metastasis. However, other, non-genomic, factors are also likely to contribute to metastasis, and more sophisticated combinations of genomic, epigenomic, transcriptomic and proteomic analyses at the single-cell level will be required to further delineate the functional implications of tumour heterogeneity during the metastatic process.

## Cell plasticity during the metastatic process

Whereas malignant transformation and tumour progression critically depend on somatic mutations, metastasis appears to largely be driven by reversible epigenetic changes. Throughout the invasion-metastasis cascade, cancer cells need to adapt to their ever-changing microenvironment, indicating that cell plasticity is essential for metastasis formation. In this context, epithelial-to-mesenchymal plasticity has been particularly well-studied, and it has been established that EMT promotes tumour-cell dissemination while MET is critical for reinitiating proliferation at the distant site and for promoting metastatic outgrowth.^101,102^ However, a more complex picture of cell plasticity and metastasis is emerging on the basis of experimental evidence, although the molecular details of how cell plasticity is defined and how specific pathways and factors contribute to EMT, cell plasticity and thus to metastasis remain to be functionally characterised (Fig. 2). The delineation of these pathways will be critical for the design of novel therapeutic strategies.

### Invasion and dissemination

Depending on the microenvironment at the primary tumour site, cancer cells migrate and invade as single cells or as cell collectives.^103^ Full EMT generates highly invasive cells that are capable of degrading the ECM by secreting proteases. These cells can disseminate as individual cells or—if multiple cells undergo EMT—as a multicellular stream. Single cells can also invade in an EMT- and protease-independent manner by amoeboid migration (Fig. 2). On the other hand, collective cell migration and invasion is characterised by the coordinated movement of cohesive groups of cells with intact cell–cell junctions.^103^ Frequently, mesenchymal leader cells at the tip of these cell collectives direct the movement and mediate degradation of the ECM. Activated stromal fibroblasts can also remodel the ECM and interact with epithelial cancer cells to facilitate invasion.^79–81^ Of note, experimental evidence suggests that activation of a partial EMT programme allows cancer cells to become motile while retaining cell–cell adhesion, thereby driving collective migration.^104–107^ Cheung and colleagues have also reported the activation of a basal epithelial programme in leader cells of collectively migrating breast cancer cells.^70,71^ These cells express E-cadherin and the basal epithelial marker cytokeratin 14 (K14) but none of the conventional mesenchymal cell markers. However, interconversion between K14^–^ and K14^+^ epithelial cell states has been observed,^70^ and these leader cells have been shown to exhibit a hybrid epithelial/mesenchymal rather than a full mesenchymal phenotype.^108^ Together with insights into the hybrid states of EMT in cancer cells, it has been concluded that specific reversible stages of the EMT continuum might confer the cell plasticity required for tumour cell dissemination and thus for metastasis formation (discussed in ref. ^47^) However, direct evidence for reversible epigenetic changes during the metastatic process in patients remains to be shown.

### Intravasation and survival in the circulation

Migratory cancer cells can eventually actively invade the blood or lymphatic vasculature as individual cells or as multicellular clusters, although, as the neovasculature of the developing tumour is abnormally permeable, a considerable number of cancer cells might also be passively shed into the circulation (Fig. 2).^109^ Once in the circulation, CTCs comprising cancer cells that have actively invaded as well as those that have been passively shed represent a heterogeneous population of cancer cells. Single CTCs expressing EMT markers have been widely observed in breast cancer patients and, in particular, the presence of CTCs with hybrid EMT phenotypes correlated with poor prognosis.^51,110,111^ Lineage labelling of cancer cells in breast and squamous cell carcinoma mouse models revealed a dramatic enrichment of cells that have undergone EMT in CTCs compared with primary tumours, indicating a strong contribution of EMT to metastatic dissemination.^48,112,113^ Intriguingly, CTC clusters also consisted of a diverse spectrum of hybrid EMT stage cancer cells as well as fully epithelial and mesenchymal cancer cells, thus supporting a role of the EMT continuum in collective cell invasion.^111^

Compared with single CTCs, multicellular CTC clusters were reported to show an up to 50-fold increased metastatic potential.^99^ This increase might be due to the physical advantage conferred by a larger size in surviving shear stress and possibly immunosurveillance in the circulation. In addition, however, CTC clusters are also able to seed polyclonal metastases, and critical molecular interactions within the cluster can define distinct cell states that are associated with the dissemination of CTC clusters.^114^ For example, hypomethylation of the promoters of the stemness regulators *OCT4*, *NANOG*, *SOX2* and *SIN3A* was observed in breast cancer CTC clusters, suggesting increased stemness of cancer cells that travel the circulation in clusters.^100^

### Metastatic outgrowth

Metastatic colonisation (Fig. 2), which involves metastatic seeding and outgrowth in distant organs, is a bottleneck for metastasis formation and requires stem cell properties. As discussed above, stemness is not a stable feature but can be acquired or lost due to cell plasticity. A plethora of studies have confirmed the crucial requirement of MET for outgrowth at the metastatic site.^101,102,115–117^ However, the plasticity and stemness associated with distinct EMT and MET states is poorly understood. To date, observations from breast, prostate and squamous cell carcinoma indicate a particularly high degree of plasticity in the intermediate EMT states, which is reflected by efficient metastatic dissemination and outgrowth.^48,118^ Notably, in a mouse model of colorectal cancer, the majority of disseminated cells in the circulation did not express Lgr5, a marker of cancer stem cells, but formed distant metastases in which Lgr5-positive cells were subsequently detected. Thus, although the cell plasticity required for metastatic dissemination does not appear to rely on cancer cell stemness, metastatic outgrowth does.^119^

In light of these findings, the spatial and temporal aspects of cell plasticity during colonisation warrant further investigation. In particular, the relationship between cancer cell plasticity and tumour dormancy is of great interest. Cells in less plastic EMT states can persist at the metastatic site for an extended period of time before eventually undergoing MET and growing out as a macrometastasis. Consistent with this notion, experimental evidence linking tumour dormancy and mesenchymal CSCs is increasing,^120^ yet needs to be further investigated, since the actual phenotypic state of dormant tumour cells still remains enigmatic. Similarly, the association of distinct EMT hybrid states and metastatic organotropism should be further explored. For instance, Reichert et al. reported that mesenchymal pancreatic ductal adenocarcinoma cells might preferentially form lung metastases over liver metastases when prevented from undergoing MET.^121^

## The relevance of EMT in metastasis and therapy resistance

Even though EMT/MET can contribute to various aspects of the invasion-metastasis cascade, the relevance of EMT in cancer metastasis is still debated. In order to track the fate of cancer cells that have undergone EMT, cell-specific lineage-tracing systems have been developed; these are based on the expression of Cre recombinase under the control of promoters of the mesenchymal marker genes fibroblast-specific protein (*Fsp*), vimentin (*Vim*) or α-smooth muscle actin (αSMA; *Acta2*) in combination with a tdTomato-to-GFP colour-switching reporter.^122,123^ Such lineage-tracing systems, combined with a variety of conditional transgenic mouse models of cancer, have been used to study the contribution of EMT to metastasis in a whole organism context (Table 1 and reviewed in ref. ^124^) Although lineage-traced mesenchymal cancer cells have been reported to be highly enriched in CTCs compared with primary tumours,^123^ only a minority of metastatic lesions were observed to be derived from cells that had undergone EMT.^51,122,123^ Furthermore, although the experimental induction of reversible EMT has been clearly demonstrated to be sufficient for promoting metastasis,^102,115^ the inhibition of EMT by tumour-cell-specific knockout of the key EMT transcription factors Snail1, Zeb1 or Twist1 in various carcinoma models has led to conflicting results.^50,51,125,126^ These inconsistencies might be explained by tissue-specific functions and overlapping, yet distinct, roles for these EMT-inducing transcription factors, thus precluding the complete inhibition of the EMT programme by the elimination of only one factor.^127^

**Table 1:** Evidence for and against a contribution of EMT to metastasis in mouse models.

*BC* breast cancer, *cKO* conditional knockout, *PC* prostate cancer, *PDAC* pancreatic ductal adenocarcinoma, *PNET* pancreatic neuroendocrine tumour, *SCC* squamous cell carcinoma.

What is clear from these experiments is that cancer cells that have undergone EMT are more refractory to conventional chemotherapies and, thus, are enriched in treated primary tumours and metastasis—indeed, EMT-traced tumour cells were highly enriched in tumours of mice treated with conventional chemotherapies.^51,123^ Based on these observations a discussion had been started whether EMT is dispensable for metastasis formation, but critical for the development of therapy resistance. However, these early studies are limited by the use of Fsp, Vim and αSMA as EMT markers—these are markers of late EMT and are not universally expressed during EMT, as well as also being expressed by and thus tracing CAFs.^113,124,128^ Most importantly, cells in an early, partial EMT state have not been marked by lineage tracing in these experiments and, thus, have not been monitored and analysed. Furthermore, it is possible that cells that have undergone (partial) EMT contribute to the metastasis of epithelial cells, perhaps by leading collective invasion strands or by promoting ECM degradation, without themselves disseminating or surviving at the metastatic site.

Based on current knowledge, the contribution of EMT to metastasis is likely to be context-dependent. Although EMT is sufficient to induce cancer cell dissemination, epithelial cells and cells undergoing partial EMT—especially when travelling as clusters—might establish metastases more efficiently than fully mesenchymal cancer cells. Consistent with this, it has been shown that the loss of E-cadherin—a hallmark of full EMT—promotes local dissemination but at the same time reduces proliferation, survival, metastatic colonisation and metastatic outgrowth.^129^ This important result is consistent with the notion that EMT is required for local cancer cell invasion, but that MET is required for metastatic outgrowth at the distant site.^47,101,102^

In summary, we need to consider that microenvironmental factors at distinct steps of the metastatic cascade could select for certain EMT states and modes of dissemination. At the same time, cell plasticity allows cancer cells to transition between EMT states. The contribution of a partial or full EMT programme to the metastatic burden might therefore critically depend on microenvironmental and cancer-cell-intrinsic factors and could dynamically change during tumour progression or during the course of therapy. Indeed, the contribution of EMT to therapy resistance has been amply demonstrated in various experimental systems (summarised in ref. ^52^) and is consistent with the observation that therapy-resistant tumours in patients frequently exhibit highly aggressive phenotypes. To dissect the molecular details that might distinguish the impact of EMT on metastasis from that on therapy resistance, we need to better understand the functional characteristics of distinct EMT states and dynamics of the transition between them. In other words, we need to define the type of cell plasticity that enables metastasis formation or therapy resistance at a molecular or omics level to identify novel therapeutic targets for preventing metastasis and/or therapy resistance.

## The clinical consequences of breast cancer heterogeneity

The heterogeneous nature of breast cancer increases a tumour’s ability to adapt to constantly changing constraints and is a significant hurdle for diagnosis and therapy.^2,39^ Due to the existence of spatial and temporal heterogeneity, it is important to mitigate the risk of sampling bias and to monitor the evolution of the disease at the entire tumour level at initial diagnosis as well as upon relapse. The comparison of multiple, spatially separated breast tumour sections together with the non-invasive analysis of CTCs and the evaluation of individual metastatic lesions throughout tumour progression will increase our understanding of intratumoural heterogeneity and hopefully improve patient diagnosis and treatment.^2,11,13,16,39,130^ This idea should be approached from various angles, most importantly by integrating genetic, epigenetic, transcriptomic, proteomic and other -omic analyses, to ultimately develop treatment strategies that can be tailored to the individual patient.^131–135^

Therapeutic regimens can target either the cancer cells themselves or the TME.^74,136,137^ Targeted therapy aimed at cancer cells carries the risk of drug resistance due to the presence of cells that do not rely on the specified target or due to adaptation processes driven by the high mutational rate or the high cell plasticity of cancer cells. By contrast, stromal cells are genetically and epigenetically more stable, which might decrease the risk of resistance and recurrence.^75,76,138^ However, due to the extensive cooperation of different subclones of cancer cells between each other and with stromal cells, the eradication of distinct cell populations or inhibition of specific cell–cell interactions might lead to unwanted adjustments in the microenvironment that could drive therapy resistance. Examples of the development of such resistance also include metabolic changes and fundamental adaptations to overcome cell death and/or immune surveillance.^139,140^

Beyond cancer-cell-intrinsic changes, the interplay between cancer cells and cells of the TME during the development of therapy resistance has just started to be addressed, and identification of adaptations in such interactions offers the opportunity to delineate the molecular pathways and processes that are critical for the survival of therapy-resistant cancer cells. The identification of an Achilles heel of therapy-resistant cancer cells is the aim of so-called synthetic lethal interference therapies, which are designed to kill only therapy-resistant cancer cells, not non-transformed cell in the body, in combination therapies. Hence, a more comprehensive understanding of the interactions between all cells within the TME and of the critical pathophysiological, biochemical and molecular parameters during malignant tumour progression and therapy response is required to develop efficacious combinatorial treatment regimens for individual patients in a personalised approach.^12,13^

The attainment of high cell plasticity enables an individual cancer cell to phenotypically adapt to microenvironmental constraints, which, in turn, fuels tumour heterogeneity and also fosters therapy resistance. Numerous studies have associated EMT with drug resistance. However, the relationships between distinct EMT states, cell plasticity and therapy resistance remain unknown.^46^ Ishay-Ronen and colleagues^54^ have exploited the plasticity associated with hybrid EMT states to transdifferentiate highly aggressive and metastatic breast cancer cells into bona fide post-mitotic adipocytes, which, in turn, resulted in a reduction of primary tumour invasion and lung metastasis formation.^54^ This study points towards the intriguing possibility of directly targeting cell plasticity for the purpose of overcoming therapy resistance. However, a better understanding of the mechanisms associated with cancer cell plasticity and its impact on tumour progression is warranted to further develop these novel treatment strategies.

## Conclusions

Despite advances in various sequencing techniques, we are still far away from translating our increasing functional and molecular understanding of tumour heterogeneity and cancer cell plasticity into clinical practice. In 2018, more than 620,000 women lost their lives to breast cancer, mostly due to the presence of tumour heterogeneity and its consequences emerging as metastasis formation and therapy resistance.^141^ This sobering concept exemplifies the clinical need to gain an in-depth understanding of tumour heterogeneity and cell plasticity during cancer progression to ultimately develop novel therapeutic strategies tailored to individual patients. In this article, we have described tumour heterogeneity at the level of the tumour cells themselves, at the level of the varying cell types which in addition to tumour cells infiltrate and constitute tumours, and at the level of the TME which, of course, is defined by the factors and molecules secreted by the cells within tumours. These multiple levels of tumour heterogeneity obviously have clinical consequences. The described polyclonal nature of breast cancer in time and space should be taken into consideration throughout clinical decision-making, particularly for patient diagnosis and treatment selection. To reduce the risk of sampling bias and to stratify patients to receive the correct therapeutic regimen, tumour heterogeneity should be evaluated by all available omics technologies by the analysis of primary tumours, of CTCs and, whenever possible, by biopsies of secondary lesions. Of course, it is currently not possible to predict how this type of comprehensive data collection will ultimately facilitate an adequate choice of therapy and improve the clinical outcome for an individual patient. However, in the course of current efforts to use available technologies for the comprehensive analysis of individual patients (frequently referred to as Personalised Health Oncology), it is important to evaluate these opportunities and to assess whether an individually selected combination therapy will finally improve patient care.

## Data Availability

Not applicable.

## References

[1] Harbeck N, Penault-Llorca F, Cortes J, Gnant M, Houssami N, Poortmans P (2019). Breast cancer. Nat. Rev. Dis. Primers.

[2] Koren S, Bentires-Alj M (2015). Breast tumor heterogeneity: source of fitness, hurdle for therapy. Mol. Cell.

[3] Melchor L, Molyneux G, Mackay A, Magnay FA, Atienza M, Kendrick H (2014). Identification of cellular and genetic drivers of breast cancer heterogeneity in genetically engineered mouse tumour models. J. Pathol..

[4] Skibinski A, Kuperwasser C (2015). The origin of breast tumor heterogeneity. Oncogene.

[5] Visvader JE, Stingl J (2014). Mammary stem cells and the differentiation hierarchy: current status and perspectives. Genes Dev..

[6] Perou CM, Sorlie T, Eisen MB, van de Rijn M, Jeffrey SS, Rees CA (2000). Molecular portraits of human breast tumours. Nature.

[7] Prat A, Parker JS, Karginova O, Fan C, Livasy C, Herschkowitz JI (2010). Phenotypic and molecular characterization of the claudin-low intrinsic subtype of breast cancer. Breast Cancer Res..

[8] Prat A, Perou CM (2011). Deconstructing the molecular portraits of breast cancer. Mol. Oncol..

[9] Ellis MJ, Perou CM (2013). The genomic landscape of breast cancer as a therapeutic roadmap. Cancer Discov..

[10] Bissell MJ, Hall HG, Parry G (1982). How does the extracellular matrix direct gene expression?. J. Theor. Biol..

[11] Bedard PL, Hansen AR, Ratain MJ, Siu LL (2013). Tumour heterogeneity in the clinic. Nature.

[12] Brooks MD, Burness ML, Wicha MS (2015). Therapeutic implications of cellular heterogeneity and plasticity in breast cancer. Cell Stem Cell.

[13] Lawson DA, Kessenbrock K, Davis RT, Pervolarakis N, Werb Z (2018). Tumour heterogeneity and metastasis at single-cell resolution. Nat. Cell. Biol..

[14] Marusyk A, Almendro V, Polyak K (2012). Intra-tumour heterogeneity: a looking glass for cancer?. Nat. Rev. Cancer.

[15] McGranahan N, Swanton C (2017). Clonal heterogeneity and tumor evolution: past, present, and the future. Cell.

[16] Alizadeh AA, Aranda V, Bardelli A, Blanpain C, Bock C, Borowski C (2015). Toward understanding and exploiting tumor heterogeneity. Nat. Med..

[17] van 't Veer LJ, Dai H, van de Vijver MJ, He YD, Hart AA, Mao M (2002). Gene expression profiling predicts clinical outcome of breast cancer. Nature.

[18] Navin N, Kendall J, Troge J, Andrews P, Rodgers L, McIndoo J (2011). Tumour evolution inferred by single-cell sequencing. Nature.

[19] Shah SP, Morin RD, Khattra J, Prentice L, Pugh T, Burleigh A (2009). Mutational evolution in a lobular breast tumour profiled at single nucleotide resolution. Nature.

[20] Curtis C, Shah SP, Chin SF, Turashvili G, Rueda OM, Dunning MJ (2012). The genomic and transcriptomic architecture of 2,000 breast tumours reveals novel subgroups. Nature.

[21] Shah SP, Roth A, Goya R, Oloumi A, Ha G, Zhao Y (2012). The clonal and mutational evolution spectrum of primary triple-negative breast cancers. Nature.

[22] Cancer Genome Atlas N (2012). Comprehensive molecular portraits of human breast tumours. Nature.

[23] Russnes HG, Lingjaerde OC, Borresen-Dale AL, Caldas C (2017). Breast cancer molecular stratification: from intrinsic subtypes to integrative clusters. Am. J. Pathol..

[24] Banerji S, Cibulskis K, Rangel-Escareno C, Brown KK, Carter SL, Frederick AM (2012). Sequence analysis of mutations and translocations across breast cancer subtypes. Nature.

[25] Greaves M, Maley CC (2012). Clonal evolution in cancer. Nature.

[26] Nowell PC (1976). The clonal evolution of tumor cell populations. Science.

[27] Campbell PJ, Yachida S, Mudie LJ, Stephens PJ, Pleasance ED, Stebbings LA (2010). The patterns and dynamics of genomic instability in metastatic pancreatic cancer. Nature.

[28] Yachida S, Jones S, Bozic I, Antal T, Leary R, Fu B (2010). Distant metastasis occurs late during the genetic evolution of pancreatic cancer. Nature.

[29] Nik-Zainal S, Alexandrov LB, Wedge DC, Van Loo P, Greenman CD, Raine K (2012). Mutational processes molding the genomes of 21 breast cancers. Cell.

[30] Nik-Zainal S, Van Loo P, Wedge DC, Alexandrov LB, Greenman CD, Lau KW (2012). The life history of 21 breast cancers. Cell.

[31] Nguyen LV, Cox CL, Eirew P, Knapp DJ, Pellacani D, Kannan N (2014). DNA barcoding reveals diverse growth kinetics of human breast tumour subclones in serially passaged xenografts. Nat. Commun..

[32] Nguyen LV, Pellacani D, Lefort S, Kannan N, Osako T, Makarem M (2015). Barcoding reveals complex clonal dynamics of de novo transformed human mammary cells. Nature.

[33] Echeverria GV, Powell E, Seth S, Ge Z, Carugo A, Bristow C (2018). High-resolution clonal mapping of multi-organ metastasis in triple negative breast cancer. Nat. Commun..

[34] Merino D, Weber TS, Serrano A, Vaillant F, Liu K, Pal B (2019). Barcoding reveals complex clonal behavior in patient-derived xenografts of metastatic triple negative breast cancer. Nat. Commun..

[35] Wagenblast E, Soto M, Gutierrez-Angel S, Hartl CA, Gable AL, Maceli AR (2015). A model of breast cancer heterogeneity reveals vascular mimicry as a driver of metastasis. Nature.

[36] Hunter KW, Amin R, Deasy S, Ha NH, Wakefield L (2018). Genetic insights into the morass of metastatic heterogeneity. Nat. Rev. Cancer.

[37] Klein CA (2013). Selection and adaptation during metastatic cancer progression. Nature.

[38] Casasent AK, Schalck A, Gao R, Sei E, Long A, Pangburn W (2018). Multiclonal invasion in breast tumors identified by topographic single cell sequencing. Cell.

[39] Swanton C (2012). Intratumor heterogeneity: evolution through space and time. Cancer Res..

[40] Yates LR, Gerstung M, Knappskog S, Desmedt C, Gundem G, Van Loo P (2015). Subclonal diversification of primary breast cancer revealed by multiregion sequencing. Nat. Med..

[41] Desmedt C, Fumagalli D, Pietri E, Zoppoli G, Brown D, Nik-Zainal S (2015). Uncovering the genomic heterogeneity of multifocal breast cancer. J. Pathol..

[42] Gupta PB, Pastushenko I, Skibinski A, Blanpain C, Kuperwasser C (2019). Phenotypic plasticity: driver of cancer initiation, progression, and therapy resistance. Cell Stem Cell.

[43] Prindull G, Zipori D (2004). Environmental guidance of normal and tumor cell plasticity: epithelial mesenchymal transitions as a paradigm. Blood.

[44] Tata PR, Rajagopal J (2016). Cellular plasticity: 1712 to the present day. Curr. Opin. Cell Biol..

[45] Nieto MA, Huang RY, Jackson RA, Thiery JP (2016). Emt: 2016. Cell.

[46] Pastushenko I, Blanpain C (2019). EMT transition states during tumor progression and metastasis. Trends Cell Biol..

[47] Yang, J., Antin, P., Berx, G., Blanpain, C., Brabletz, T., Bronner, M. et al. Guidelines and definitions for research on epithelial-mesenchymal transition. *Nat. Rev. Mol. Cell Biol*. 10.1038/s41580-020-0237-9 (2020). 10.1038/s41580-020-0237-9 PMC7250738 32300252

[48] Pastushenko I, Brisebarre A, Sifrim A, Fioramonti M, Revenco T, Boumahdi S (2018). Identification of the tumour transition states occurring during EMT. Nature.

[49] Rios AC, Capaldo BD, Vaillant F, Pal B, van Ineveld R, Dawson CA (2019). Intraclonal plasticity in mammary tumors revealed through large-scale single-cell resolution 3D imaging. Cancer Cell.

[50] Krebs AM, Mitschke J, Lasierra Losada M, Schmalhofer O, Boerries M, Busch H (2017). The EMT-activator Zeb1 is a key factor for cell plasticity and promotes metastasis in pancreatic cancer. Nat. Cell Biol..

[51] Zheng X, Carstens JL, Kim J, Scheible M, Kaye J, Sugimoto H (2015). Epithelial-to-mesenchymal transition is dispensable for metastasis but induces chemoresistance in pancreatic cancer. Nature.

[52] Brabletz T, Kalluri R, Nieto MA, Weinberg RA (2018). EMT in cancer. Nat. Rev. Cancer.

[53] Lawson DA, Bhakta NR, Kessenbrock K, Prummel KD, Yu Y, Takai K (2015). Single-cell analysis reveals a stem-cell program in human metastatic breast cancer cells. Nature.

[54] Ishay-Ronen D, Diepenbruck M, Kalathur RKR, Sugiyama N, Tiede S, Ivanek R (2019). Gain fat-lose metastasis: converting invasive breast cancer cells into adipocytes inhibits cancer metastasis. Cancer Cell.

[55] Mani SA, Guo W, Liao MJ, Eaton EN, Ayyanan A, Zhou AY (2008). The epithelial-mesenchymal transition generates cells with properties of stem cells. Cell.

[56] Wellner U, Schubert J, Burk UC, Schmalhofer O, Zhu F, Sonntag A (2009). The EMT-activator ZEB1 promotes tumorigenicity by repressing stemness-inhibiting microRNAs. Nat. Cell Biol..

[57] Ye X, Tam WL, Shibue T, Kaygusuz Y, Reinhardt F, Ng Eaton E (2015). Distinct EMT programs control normal mammary stem cells and tumour-initiating cells. Nature.

[58] Chaffer CL, Marjanovic ND, Lee T, Bell G, Kleer CG, Reinhardt F (2013). Poised chromatin at the ZEB1 promoter enables breast cancer cell plasticity and enhances tumorigenicity. Cell.

[59] Gupta PB, Fillmore CM, Jiang G, Shapira SD, Tao K, Kuperwasser C (2011). Stochastic state transitions give rise to phenotypic equilibrium in populations of cancer cells. Cell.

[60] Klevebring D, Rosin G, Ma R, Lindberg J, Czene K, Kere J (2014). Sequencing of breast cancer stem cell populations indicates a dynamic conversion between differentiation states in vivo. Breast Cancer Res..

[61] Granit RZ, Masury H, Condiotti R, Fixler Y, Gabai Y, Glikman T (2018). Regulation of cellular heterogeneity and rates of symmetric and asymmetric divisions in triple-negative breast cancer. Cell Rep..

[62] Koren S, Reavie L, Couto JP, De Silva D, Stadler MB, Roloff T (2015). PIK3CA(H1047R) induces multipotency and multi-lineage mammary tumours. Nature.

[63] Van Keymeulen A, Lee MY, Ousset M, Brohee S, Rorive S, Giraddi RR (2015). Reactivation of multipotency by oncogenic PIK3CA induces breast tumour heterogeneity. Nature.

[64] Trimboli AJ, Fukino K, de Bruin A, Wei G, Shen L, Tanner SM (2008). Direct evidence for epithelial-mesenchymal transitions in breast cancer. Cancer Res..

[65] Kitamura T, Qian BZ, Pollard JW (2015). Immune cell promotion of metastasis. Nat. Rev. Immunol..

[66] Cleary AS, Leonard TL, Gestl SA, Gunther EJ (2014). Tumour cell heterogeneity maintained by cooperating subclones in Wnt-driven mammary cancers. Nature.

[67] Polyak K, Marusyk A (2014). Cancer: clonal cooperation. Nature.

[68] Kim MY, Oskarsson T, Acharyya S, Nguyen DX, Zhang XH, Norton L (2009). Tumor self-seeding by circulating cancer cells. Cell.

[69] Gundem G, Van Loo P, Kremeyer B, Alexandrov LB, Tubio JMC, Papaemmanuil E (2015). The evolutionary history of lethal metastatic prostate cancer. Nature.

[70] Cheung KJ, Padmanaban V, Silvestri V, Schipper K, Cohen JD, Fairchild AN (2016). Polyclonal breast cancer metastases arise from collective dissemination of keratin 14-expressing tumor cell clusters. Proc. Natl Acad. Sci. USA.

[71] Cheung KJ, Gabrielson E, Werb Z, Ewald AJ (2013). Collective invasion in breast cancer requires a conserved basal epithelial program. Cell.

[72] Archetti M, Pienta KJ (2019). Cooperation among cancer cells: applying game theory to cancer. Nat. Rev. Cancer.

[73] Joyce JA, Pollard JW (2009). Microenvironmental regulation of metastasis. Nat. Rev. Cancer.

[74] Junttila MR, de Sauvage FJ (2013). Influence of tumour micro-environment heterogeneity on therapeutic response. Nature.

[75] Quail DF, Joyce JA (2013). Microenvironmental regulation of tumor progression and metastasis. Nat. Med..

[76] Tabassum DP, Polyak K (2015). Tumorigenesis: it takes a village. Nat. Rev. Cancer.

[77] Marusyk A, Tabassum DP, Altrock PM, Almendro V, Michor F, Polyak K (2014). Non-cell-autonomous driving of tumour growth supports sub-clonal heterogeneity. Nature.

[78] Goetz JG, Minguet S, Navarro-Lerida I, Lazcano JJ, Samaniego R, Calvo E (2011). Biomechanical remodeling of the microenvironment by stromal caveolin-1 favors tumor invasion and metastasis. Cell.

[79] Gaggioli C, Hooper S, Hidalgo-Carcedo C, Grosse R, Marshall JF, Harrington K (2007). Fibroblast-led collective invasion of carcinoma cells with differing roles for RhoGTPases in leading and following cells. Nat. Cell Biol..

[80] Dang TT, Prechtl AM, Pearson GW (2011). Breast cancer subtype-specific interactions with the microenvironment dictate mechanisms of invasion. Cancer Res..

[81] Matise LA, Palmer TD, Ashby WJ, Nashabi A, Chytil A, Aakre M (2012). Lack of transforming growth factor-beta signaling promotes collective cancer cell invasion through tumor-stromal crosstalk. Breast Cancer Res..

[82] Malanchi I, Santamaria-Martinez A, Susanto E, Peng H, Lehr HA, Delaloye JF (2011). Interactions between cancer stem cells and their niche govern metastatic colonization. Nature.

[83] Balkwill F (2004). Cancer and the chemokine network. Nat. Rev. Cancer.

[84] Su S, Liu Q, Chen J, Chen J, Chen F, He C (2014). A positive feedback loop between mesenchymal-like cancer cells and macrophages is essential to breast cancer metastasis. Cancer Cell.

[85] Lewis CE, Harney AS, Pollard JW (2016). The multifaceted role of perivascular macrophages in tumors. Cancer Cell.

[86] Linde N, Casanova-Acebes M, Sosa MS, Mortha A, Rahman A, Farias E (2018). Macrophages orchestrate breast cancer early dissemination and metastasis. Nat. Commun..

[87] Arwert EN, Harney AS, Entenberg D, Wang Y, Sahai E, Pollard JW (2018). A unidirectional transition from migratory to perivascular macrophage is required for tumor cell intravasation. Cell Rep..

[88] Janiszewska M, Tabassum DP, Castano Z, Cristea S, Yamamoto KN, Kingston NL (2019). Subclonal cooperation drives metastasis by modulating local and systemic immune microenvironments. Nat. Cell Biol..

[89] Ombrato L, Malanchi I (2019). Subclonal cooperation rewrites metastasis. Nat. Cell Biol..

[90] Szczerba BM, Castro-Giner F, Vetter M, Krol I, Gkountela S, Landin J (2019). Neutrophils escort circulating tumour cells to enable cell cycle progression. Nature.

[91] Mohme M, Riethdorf S, Pantel K (2017). Circulating and disseminated tumour cells—mechanisms of immune surveillance and escape. Nat. Rev. Clin. Oncol..

[92] Jiang Y, Zhan H (2020). Communication between EMT and PD-L1 signaling: new insights into tumor immune evasion. Cancer Lett..

[93] Kalbasi A, Ribas A (2020). Tumour-intrinsic resistance to immune checkpoint blockade. Nat. Rev. Immunol..

[94] Gilkes DM, Semenza GL, Wirtz D (2014). Hypoxia and the extracellular matrix: drivers of tumour metastasis. Nat. Rev. Cancer.

[95] Kang Y, Siegel PM, Shu W, Drobnjak M, Kakonen SM, Cordón-Cardo C (2003). A multigenic program mediating breast cancer metastasis to bone. Cancer Cell.

[96] Minn AJ, Gupta GP, Siegel PM, Bos PD, Shu W, Giri DD (2005). Genes that mediate breast cancer metastasis to lung. Nature.

[97] Hu Z, Li Z, Ma Z, Curtis C (2020). Multi-cancer analysis of clonality and the timing of systemic spread in paired primary tumors and metastases. Nat. Genet..

[98] Obradovic MMS, Hamelin B, Manevski N, Couto JP, Sethi A, Coissieux MM (2019). Glucocorticoids promote breast cancer metastasis. Nature.

[99] Aceto N, Bardia A, Miyamoto DT, Donaldson MC, Wittner BS, Spencer JA (2014). Circulating tumor cell clusters are oligoclonal precursors of breast cancer metastasis. Cell.

[100] Gkountela S, Castro-Giner F, Szczerba BM, Vetter M, Landin J, Scherrer R (2019). Circulating tumor cell clustering shapes DNA methylation to enable metastasis seeding. Cell.

[101] Ocana OH, Corcoles R, Fabra A, Moreno-Bueno G, Acloque H, Vega S (2012). Metastatic colonization requires the repression of the epithelial-mesenchymal transition inducer Prrx1. Cancer Cell.

[102] Tsai JH, Donaher JL, Murphy DA, Chau S, Yang J (2012). Spatiotemporal regulation of epithelial-mesenchymal transition is essential for squamous cell carcinoma metastasis. Cancer Cell.

[103] Friedl P, Alexander S (2011). Cancer invasion and the microenvironment: plasticity and reciprocity. Cell.

[104] Campbell K, Rossi F, Adams J, Pitsidianaki I, Barriga FM, Garcia-Gerique L (2019). Collective cell migration and metastases induced by an epithelial-to-mesenchymal transition in Drosophila intestinal tumors. Nat. Commun..

[105] Bocci, F., Jolly, M. K., Tripathi, S. C., Aguilar, M., Hanash, S. M., Levine, H. et al. Numb prevents a complete epithelial-mesenchymal transition by modulating Notch signalling. *J. R. Soc. Interface* **14**, 20170512 (2017). 10.1098/rsif.2017.0512 PMC5721160 29187638

[106] Dang TT, Esparza MA, Maine EA, Westcott JM, Pearson GW (2015). DeltaNp63alpha promotes breast cancer cell motility through the selective activation of components of the epithelial-to-mesenchymal transition program. Cancer Res..

[107] Aiello NM, Maddipati R, Norgard RJ, Balli D, Li J, Yuan S (2018). EMT subtype influences epithelial plasticity and mode of cell migration. Dev. Cell.

[108] Yang C, Cao M, Liu Y, He Y, Du Y, Zhang G (2019). Inducible formation of leader cells driven by CD44 switching gives rise to collective invasion and metastases in luminal breast carcinomas. Oncogene.

[109] Bockhorn M, Jain RK, Munn LL (2007). Active versus passive mechanisms in metastasis: do cancer cells crawl into vessels, or are they pushed?. Lancet Oncol..

[110] Polioudaki H, Agelaki S, Chiotaki R, Politaki E, Mavroudis D, Matikas A (2015). Variable expression levels of keratin and vimentin reveal differential EMT status of circulating tumor cells and correlation with clinical characteristics and outcome of patients with metastatic breast cancer. BMC Cancer.

[111] Yu M, Bardia A, Wittner BS, Stott SL, Smas ME, Ting DT (2013). Circulating breast tumor cells exhibit dynamic changes in epithelial and mesenchymal composition. Science.

[112] Beerling E, Seinstra D, de Wit E, Kester L, van der Velden D, Maynard C (2016). Plasticity between epithelial and mesenchymal states unlinks EMT from metastasis-enhancing stem cell capacity. Cell Rep..

[113] Bornes L, van Scheppingen RH, Beerling E, Schelfhorst T, Ellenbroek SIJ, Seinstra D (2019). Fsp1-mediated lineage tracing fails to detect the majority of disseminating cells undergoing EMT. Cell Rep..

[114] Castro-Giner F, Aceto N (2020). Tracking cancer progression: from circulating tumor cells to metastasis. Genome Med..

[115] Tran HD, Luitel K, Kim M, Zhang K, Longmore GD, Tran DD (2014). Transient SNAIL1 expression is necessary for metastatic competence in breast cancer. Cancer Res..

[116] Stankic M, Pavlovic S, Chin Y, Brogi E, Padua D, Norton L (2013). TGF-beta-Id1 signaling opposes Twist1 and promotes metastatic colonization via a mesenchymal-to-epithelial transition. Cell Rep..

[117] Del Pozo Martin Y, Park D, Ramachandran A, Ombrato L, Calvo F, Chakravarty P (2015). Mesenchymal cancer cell-stroma crosstalk promotes niche activation, epithelial reversion, and metastatic colonization. Cell Rep..

[118] Ruscetti M, Quach B, Dadashian EL, Mulholland DJ, Wu H (2015). Tracking and functional characterization of epithelial-mesenchymal transition and mesenchymal tumor cells during prostate cancer metastasis. Cancer Res..

[119] Fumagalli A, Oost KC, Kester L, Morgner J, Bornes L, Bruens L (2020). Plasticity of Lgr5-negative cancer cells drives metastasis in colorectal cancer. Cell Stem Cell.

[120] Weidenfeld K, Barkan D (2018). EMT and stemness in tumor dormancy and outgrowth: are they intertwined processes?. Front. Oncol..

[121] Reichert M, Bakir B, Moreira L, Pitarresi JR, Feldmann K, Simon L (2018). Regulation of epithelial plasticity determines metastatic organotropism in pancreatic cancer. Dev. Cell.

[122] Chen, Y., LeBleu, V. S., Carstens, J. L., Sugimoto, H., Zheng, X., Malasi, S. et al. Dual reporter genetic mouse models of pancreatic cancer identify an epithelial-to-mesenchymal transition-independent metastasis program. *EMBO Mol. Med*. **10**, (2018). 10.15252/emmm.201809085 PMC6180301 30120146

[123] Fischer KR, Durrans A, Lee S, Sheng J, Li F, Wong ST (2015). Epithelial-to-mesenchymal transition is not required for lung metastasis but contributes to chemoresistance. Nature.

[124] Aiello NM, Kang Y (2019). Context-dependent EMT programs in cancer metastasis. J. Exp. Med..

[125] Title AC, Hong SJ, Pires ND, Hasenohrl L, Godbersen S, Stokar-Regenscheit N (2018). Genetic dissection of the miR-200-Zeb1 axis reveals its importance in tumor differentiation and invasion. Nat. Commun..

[126] Xu Y, Lee DK, Feng Z, Xu Y, Bu W, Li Y (2017). Breast tumor cell-specific knockout of Twist1 inhibits cancer cell plasticity, dissemination, and lung metastasis in mice. Proc. Natl Acad. Sci. USA.

[127] Stemmler MP, Eccles RL, Brabletz S, Brabletz T (2019). Non-redundant functions of EMT transcription factors. Nat. Cell Biol..

[128] Ye X, Brabletz T, Kang Y, Longmore GD, Nieto MA, Stanger BZ (2017). Upholding a role for EMT in breast cancer metastasis. Nature.

[129] Padmanaban, V., Krol, I., Suhail, Y., Szczerba, B. M., Aceto, N., Bader, J. S. et al. E-cadherin is required for metastasis in multiple models of breast cancer. *Nature*10.1038/s41586-019-1526-3 (2019). 10.1038/s41586-019-1526-3 PMC7365572 31485072

[130] Potts SJ, Krueger JS, Landis ND, Eberhard DA, Young GD, Schmechel SC (2012). Evaluating tumor heterogeneity in immunohistochemistry-stained breast cancer tissue. Lab. Investig..

[131] Chen J, Suo S, Tam PP, Han JJ, Peng G, Jing N (2017). Spatial transcriptomic analysis of cryosectioned tissue samples with Geo-seq. Nat. Protoc..

[132] Macaulay IC, Haerty W, Kumar P, Li YI, Hu TX, Teng MJ (2015). G&T-seq: parallel sequencing of single-cell genomes and transcriptomes. Nat. Methods.

[133] Macaulay IC, Teng MJ, Haerty W, Kumar P, Ponting CP, Voet T (2016). Separation and parallel sequencing of the genomes and transcriptomes of single cells using G&T-seq. Nat. Protoc..

[134] Picelli S, Bjorklund AK, Faridani OR, Sagasser S, Winberg G, Sandberg R (2013). Smart-seq2 for sensitive full-length transcriptome profiling in single cells. Nat. Methods.

[135] Picelli S, Faridani OR, Bjorklund AK, Winberg G, Sagasser S, Sandberg R (2014). Full-length RNA-seq from single cells using Smart-seq2. Nat. Protoc..

[136] Paget S (1889). The distribution of secondary growths in cancer of the breast.. Cancer Metastasis Rev..

[137] Valastyan S, Weinberg RA (2011). Tumor metastasis: molecular insights and evolving paradigms. Cell.

[138] Polzer B, Klein CA (2013). Metastasis awakening: the challenges of targeting minimal residual cancer. Nat. Med..

[139] Shaked Y (2019). The pro-tumorigenic host response to cancer therapies. Nat. Rev. Cancer.

[140] Friedmann Angeli JP, Krysko DV, Conrad M (2019). Ferroptosis at the crossroads of cancer-acquired drug resistance and immune evasion. Nat. Rev. Cancer.

[141] Hanahan D, Weinberg RA (2011). Hallmarks of cancer: the next generation. Cell.

